# SCRIPT: Stratified clinical risk prediction from pathology reports using large language models

**DOI:** 10.1016/j.jpi.2026.100673

**Published:** 2026-05-12

**Authors:** Chiara M.L. Loeffler, Nic G. Reitsam, Fabian Wolf, Esther H. Stueker, Hannah S. Muti, Isabella C. Wiest, Jakob Nikolas Kather

**Affiliations:** a Else Kroener Fresenius Center for Digital Health, Faculty of Medicine and University Hospital Carl Gustav Carus, TUD Dresden University of Technology, 01307 Dresden, Germany; b Department of Medicine I, Faculty of Medicine and University Hospital Carl Gustav Carus, TUD Dresden University of Technology, 01307 Dresden, Germany; c National Center for Tumor Diseases Dresden (NCT/UCC), a partnership between DKFZ, Faculty of Medicine and University Hospital Carl Gustav Carus, TUD Dresden University of Technology, and Helmholtz-Zentrum Dresden - Rossendorf (HZDR), Dresden, Germany; d Pathology, Faculty of Medicine, University of Augsburg, Augsburg, Germany; e Bavarian Center for Cancer Research, BZKF, Augsburg, Germany; f Department for Visceral, Thoracic and Vascular Surgery, University Hospital and Faculty of Medicine Carl Gustav Carus, Technische Universität Dresden, Dresden, Germany; g Department of Medicine II, Medical Faculty Mannheim, Heidelberg University, Mannheim, Germany; h Medical Oncology, National Center for Tumor Diseases (NCT), University Hospital Heidelberg, Heidelberg, Germany; i Pathology & Data Analytics, Leeds Institute of Medical Research at St James's, University of Leeds, Leeds, United Kingdom

**Keywords:** LLM, Deep learning, Gastrointestinal cancer, Prognostic biomarker, Pathology reports

## Abstract

**Background:**

Accurate risk stratification in oncology is essential for guiding treatment decisions, yet current algorithms rely on a narrow set of structured variables, and hence potentially ignore the rich signal in narrative pathology reports. These reports contain nuanced morphological descriptions and expert clinical judgmentThis narrative information remains largely unused in clinical decision-making as it gets lost in “prose” text-based reports. We hypothesized that large language models (LLMs) could extract prognostic information from complete free-text pathology reports and convert it into a binary survival biomarker.

**Methods:**

We used the open-weight LLaMA 3.3 70B model to generate risk scores directly from publicly available pathology reports across three gastrointestinal cancer types. The model was prompted to synthesize the complete narrative reports into a binary prognostic score. We evaluated associations between the LLM-generated scores and survival outcomes, including overall survival, progression-free survival, and disease-specific survival.

**Results:**

In colorectal cancer, LLM-generated risk scores demonstrated significant prognostic value for overall survival (Hazard ratio (HR) = 2.77, 95% confidence interval (CI) = 1.92–3.97, *p* < 0.001), progression-free survival (HR = 2.93, 95% CI = 2.11–4.08, *p* < 0.001), and disease-specific survival (HR = 5.85, 95% CI = 3.66–9.36, *p* < 0.001). Multivariate analysis confirmed the LLM-generated risk score as an independent prognostic factor for progression-free survival.

**Conclusion:**

LLMs can turn narrative pathology reports into a single, independent survival biomarker. This approach leverages routinely available free-text documentation without requiring additional tissue analysis or pathologist workload, providing a deployable method to enhance risk stratification for treatment decision-making.

## Introduction

Accurately assessing the survival risk of tumor patients is crucial for guiding treatment decisions and optimizing resource allocation.^1^ Traditional risk prediction models rely on structured variables such as TNM stage or tumor grade,^2^, ^3^ yet these capture only a fraction of the biological heterogeneity reported by pathologists, leading to substantial prognostic variation within the same stage groups.^4^ Over the past decade, standardized reporting has become central to oncological pathology, ensuring that key prognostic elements such as depth of invasion, nodal status, tumor budding, and microsatellite instability (MSI) are consistently documented.^5^ However, pathology reports often retain a hybrid structure that combines narrative macro- or microscopic description with partially synoptic elements. Therefore, pathologists invest substantial time drafting detailed narrative reports, often reflecting intuitive assessments of tumor behavior based on morphological features that resist simple categorization. This represents a waste of expert knowledge: prognostic information embedded in narrative text often remains underutilized for risk stratification.

Large language models (LLMs) offer a solution by processing complete free-text pathology reports, including both narrative and synoptic components, and synthesizing them into binary risk scores. Unlike current approaches limited to manual curation or machine learning models that struggle with negation and contextual reasoning,^6^, ^7^ LLMs, such as GPT or LLaMA, have demonstrated the ability to process unstructured clinical text and support risk prediction tasks.^8^ Previous studies have explored their potential for predicting hospital mortality, intensive care unit (ICU) admission, and oncology patient triage based on unstructured health records.^9^, ^10^ For example, ChatGPT has been used to enhance triage decisions for metastatic prostate cancer patients in emergency settings.^9^ Similarly, LLMs have been applied to binary risk stratification for colon cancer patients, but these models relied on structured input data, requiring preprocessing steps that limit real-world applicability.^11^ Other studies have shown that LLMs can achieve competitive performance in predicting survival outcomes and postoperative complications for lung cancer patients using structured clinical data.^12^ Whereas these findings highlight the potential of LLMs in oncology risk prediction, their ability to directly analyze pathology reports to provide prognostic information that complements detailed staging and validated clinicopathological features remains underexplored. Our study addresses this gap by investigating whether risk scores generated by an LLM from raw pathology reports correlate with survival metrics such as overall survival (OS), progression-free survival (PFS), and disease-specific survival (DSS).

Prior work has demonstrated that retrieval-augmented generation (RAG)-enhanced LLMs can accurately extract clinically meaningful information from unstructured medical text, achieving accuracy and specificity comparable to or exceeding that of human experts.^13^

This shows that LLMs can accurately process complex, mostly unstructured clinical texts, directly supporting our approach to generate a binary prognostic risk score for gastrointestinal tumors from pathology reports. Unlike previous studies that used proprietary models requiring data anonymization and preprocessing for secure data handling, we leverage LLaMA 3.3 70B, a locally deployable LLM, to ensure privacy-preserving risk prediction. By evaluating three gastrointestinal cancer entities within TCGA, we aim to provide a novel systematic demonstration that an open-source locally deployable LLM can derive prognostic information directly from pathology reports in a zero-shot manner.

## Methods and materials

### Data acquisition and processing

**Fig. 1:**
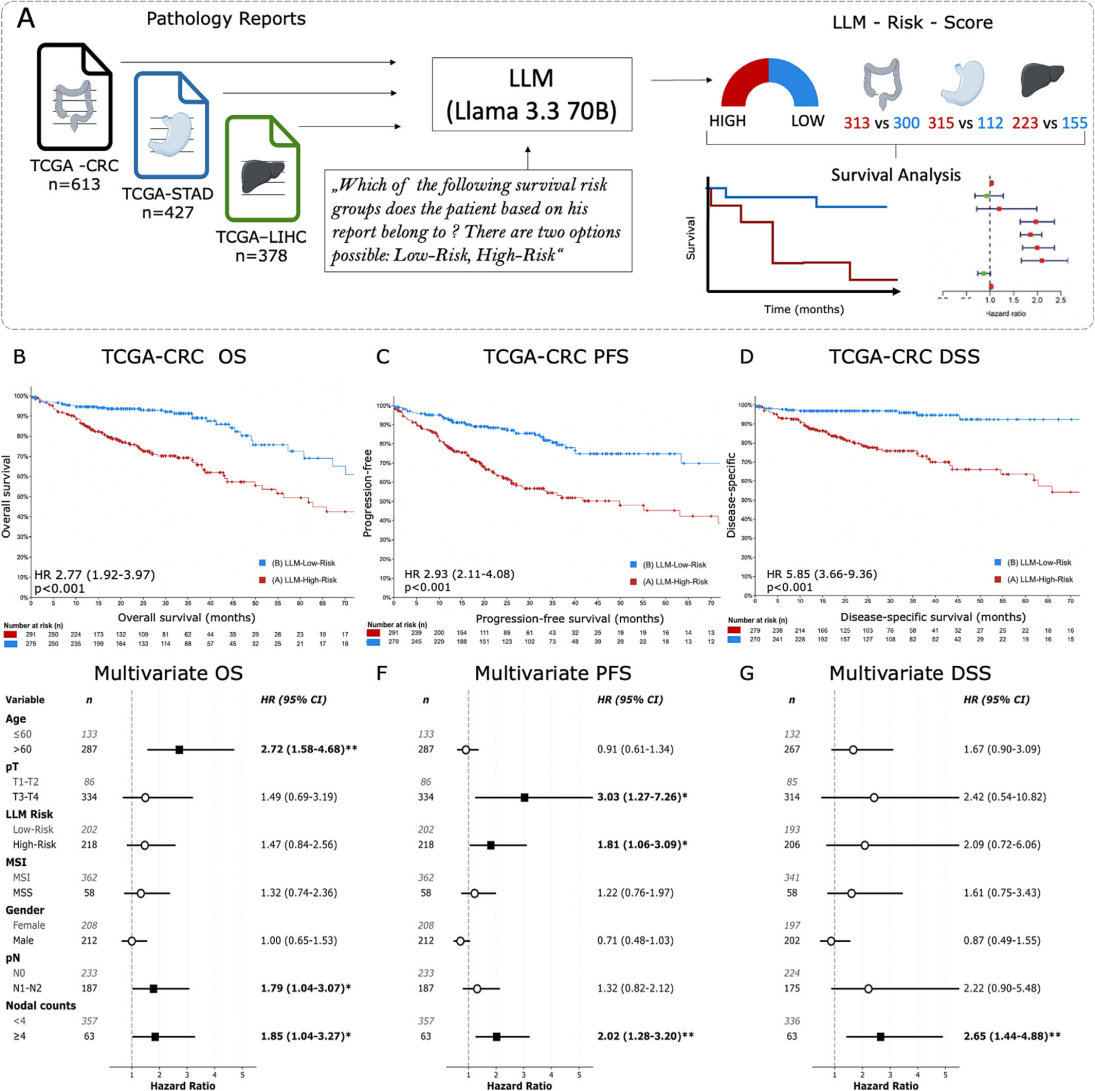
LLM-risk score can predict survival in colorectal cancer. (A) Experimental design: Pathology reports from colorectal cancer (CRC), stomach adenocarcinoma (STAD), and liver hepatocellular carcinoma (LIHC), and system prompt were fed into the large language model LLaMA v. 3.3. Output was a binary categorical value either high- or low-risk. Survival analysis with Kaplan–Meier estimator and Cox proportional hazard models was performed. Kaplan–Meier curves and Forest plot showing multivariate Cox regression analysis for (B) overall survival (OS), (C) progression-free survival (PFS), and (D) disease-specific survival (DSS) stratified by LLM high-risk and LLM low-risk patients. Covariates included age, gender, LLM risk score, pathological T-Stage (pT), pathological N-Stage (pN), positive nodal count, and MSI status. HR and 95% CI were calculated by the Cox proportional hazard model. *P*-value was calculated using the two-sided log-rank test (*p < 0.05, **p < 0.001). Plots were generated using the lifelines package in Python 3.11.5 and cbioportal.org. TCGA: The Cancer Genome Atlas, LLaMA: large language model meta AI, LLM: large language model, MSI = microsatellite instability, MSS = microsatellite stability, HR = hazard ratio, CI = confidence interval.

**Supplementary figure 1:**
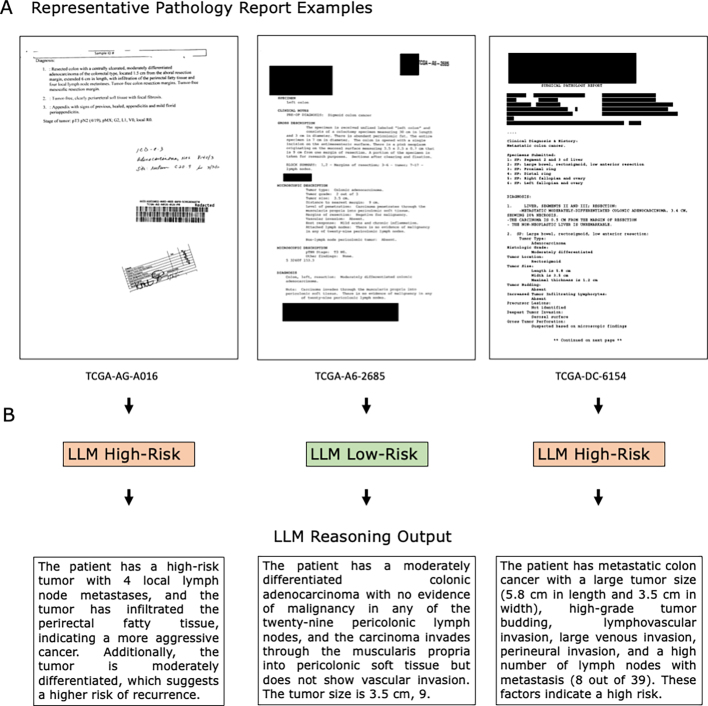
Representative Pathology Reports and Corresponding LLM Risk Assessments.(A) Examples of original pathology reports from the TCGA colorectal (CRC) cohort (e.g., TCGA-AG-A016, TCGA-A6-2685, and TCGA-DC-6154) utilized as input data. (B) Corresponding LLM outputs illustrating the classification process. The model assigns a risk category (High-Risk or Low-Risk) and generates a natural language Reasoning Output that synthesizes key clinical features.

In this study, we used *n* = 1418 pathology reports from three different gastrointestinal tumor types of The Cancer Genome Atlas (Fig. 1A). The tumor types included colorectal cancer (CRC), stomach adenocarcinoma (STAD), and liver hepatocellular carcinoma (LIHC). Pathology reports are publicly available and can be downloaded at https://portal.gdc.cancer.gov/ (accessed 12.10.2024), some random chosen examples can be found in Supplementary Fig. 1A. The reports were downloaded in PDF format, and text was extracted using optical character recognition (OCR) tools and converted into CSV format. Only reports that were successfully converted without OCR errors were retained for downstream processing. Minimal preprocessing was performed to remove headers, footers, and formatting artifacts; the semantic content of the original reports was preserved. After preprocessing, we fed the smaller text sections into the LLaMA-3.3 model containing 70 billion parameters (70B). The final text inputs varied in length, ranging from approximately 70 to 500 words. The final model output contained the binary LLM risk score and the reasoning field (Supplementary Fig. 1B and Supplementary Table 1). A detailed description of the methods has been published previously.^14^ To better define the composition of the TCGA pathology reports, we systematically screened the report text using our Information extraction pipeline^14^ for key clinicopathological concepts. For each report, we recorded whether the text contained explicit mention of: pathological T-stage (pT), pathological N-Stage (pN), pathological M-stage (pM), tumor budding, lymphovascular invasion (LVI), perineural invasion (PNI), surgical margin status, MSI/MMR status, EBV status, and lymph node counts total and positive. We then calculated the proportion of reports in which each item was present at least once (Table 1).

**Table 1:** Distribution and reporting frequency of key prognostic features in unstructured TCGA pathology reports.

Features	TCGA CRC	TCGA STAD	TCGA LIHC
pT	99.8% (581/582)	100% (411/411)	93.4% (337/361)
pN	99.8% (581/582)	100.0% (411/411)	85.0% (307/361)
pM	100.0% (582/582)	100.0% (411/411)	100.0% (361/361)
tumor budding	0.2% (1/582)	0.0% (0/411)	0.0% (0/361)
Lymphovascular invasion (LVI)	9.5% (55/582)	6.3% (26/411)	2.2% (8/361)
Perineural invasion (PNI)	1.7% (10/582)	2.9% (12/411)	0.3% (1/361)
Margin status	53.8% (313/582)	47.0% (193/411)	33.5% (121/361)
MSI/MMR	4.1% (24/582)	1.7% (7/411)	0.0% (0/361)
EBV	0.0% (0/582)	2.4% (10/411)	0.0% (0/361)

Pathology reports were systematically screened for explicit mention of key clinicopathological features using an automated information extraction pipeline. Values represent the percentage and absolute number (in parentheses) of reports in which each feature was documented at least once. CRC = colorectal cancer; STAD = stomach adenocarcinoma; LIHC = liver hepatocellular carcinoma; pT = pathological T-stage; pN = pathological N-stage; pM = pathological M-stage; LVI = lymphovascular invasion; PNI = perineural invasion; MSI = microsatellite instability; MMR = mismatch repair; EBV = Epstein-Barr virus.

### Experimental design

In this study, we used an LLM to generate a survival risk score based on tumor-specific pathology reports. First, we input the pathology reports for each tumor separately to the LLM. The user/system prompt was as follows: “*Which of the following survival risk groups does the patient based on his report belong to? There are two options possible: low-risk, high-risk”* (Fig. 1A).

The model was instructed to return structured output in JSON format with two fields:

• “risk_group”: a binary classification as either “low-risk” or “high-risk”,
• “reasoning”: a free-text rationale for the assigned risk group.

**Fig. 2:**
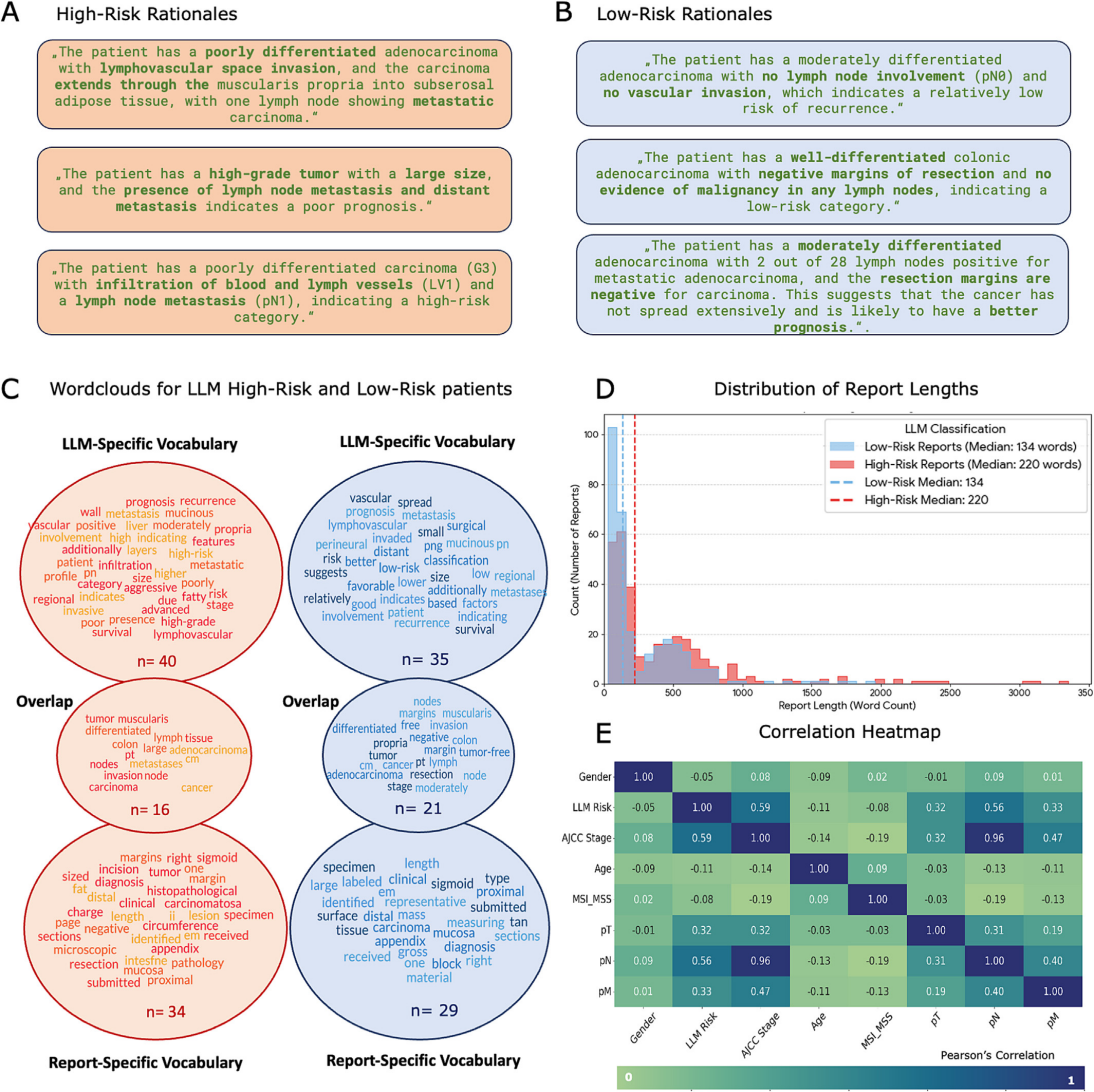
Characterization of LLM high-risk and low-risk classifications. Representative free-text rationales generated by the LLM for: (A) high-risk and (B) low-risk CRC cases. (C) LLM-specific and report-specific vocabulary, along with their overlapping vocabulary, for the LLM high-risk and low-risk groups. Wordcloud was generated using: https://www.freewordcloudgenerator.com/, (D) Histogram showing the comparison of the distribution of report lengths between the two risk groups. (E) Heatmap showing correlation between clinical variables: gender, LLM risk score, AJCC stage, age, MSI, pathological T-stage, pathological N-stage, and pathological M-stage. Barchart was created using Flourish (https://flourish.studio/). Histogram and Heatmap were generated using seaborn in Python 3.11.5.

Survival data, including OS, PFS, and DSS, were obtained from TCGA (www.cbioportal.org, accessed Nov 2024). Due to clinical data availability in cBioPortal, the study proceeded only with patients who possessed both a pathology report and complete clinical metadata. Patients were stratified into high-risk and low-risk groups based on LLM predictions. Kaplan–Meier survival curves were constructed for each survival metric, and differences were evaluated using the log-rank test. Hazard ratios (HRs) and 95% confidence intervals (CIs) were estimated using univariate and multivariate Cox proportional hazards models. Multivariate analysis was conducted using Cox proportional hazard models, including gender, age, MSI-status, pT, pN, and positive nodal count as covariates (Fig. 1). We computed the updated C-index and compared models using the likelihood-ratio test to quantify the incremental prognostic value of the LLM score. To further investigate how the LLM made its risk assignments, we analyzed representative high-risk and low-risk rationales, highlighting phrases that map to known concepts (Fig. 2A–B). Furthermore, we quantified the most frequent descriptors within the model's reasoning fields across both risk categories to identify consistent prognostic patterns. Frequency bar charts were created to show the 10 most frequently used words and their relative frequency in % in the LLM reasoning for the high-risk and low-risk groups. These 10 words were each compared with the relative frequency of the same words in the original reports of both categories. To further highlight distinctive linguistic patterns, the words that appeared in the LLM high-risk group and occurred either exclusively in the original report, exclusively in the LLM reasoning, or in both sources were identified and grouped accordingly. The same procedure was applied to the LLM low-risk group. All categories are presented in Fig. 2C. A list of the 50 most frequently used words across all categories is also provided in Supplementary Table 2. Next, we utilized the LLM-defined 300 CRC low-risk and 313 CRC high-risk pathological reports to analyze the distribution of the original report text lengths. A histogram for the comparison of the distribution of report lengths measured by words of high- and low-risk groups is found in Fig. 2D. Furthermore, a correlation heatmap was computed to assess the relationship (Pearson's correlation) between LLM predictions and clinicopathological variables using Python version 3.10.18 (Fig. 2E).

### Data and code availability

All model inference was performed on a secure local server using the Hugging Face implementation of LLaMA 3.3 70B. No patient-identifiable information was accessed or stored during the analysis. All code for model prompting, data processing, and survival analysis is available at https://github.com/KatherLab/LLMAIx under a Creative Commons Attribution 4.0 International License for full reproducibility. All software packages require a minimum of Python 3.12.

## Results

### LLM-risk score can predict survival in colorectal cancer

**Supplementary figure 2:**
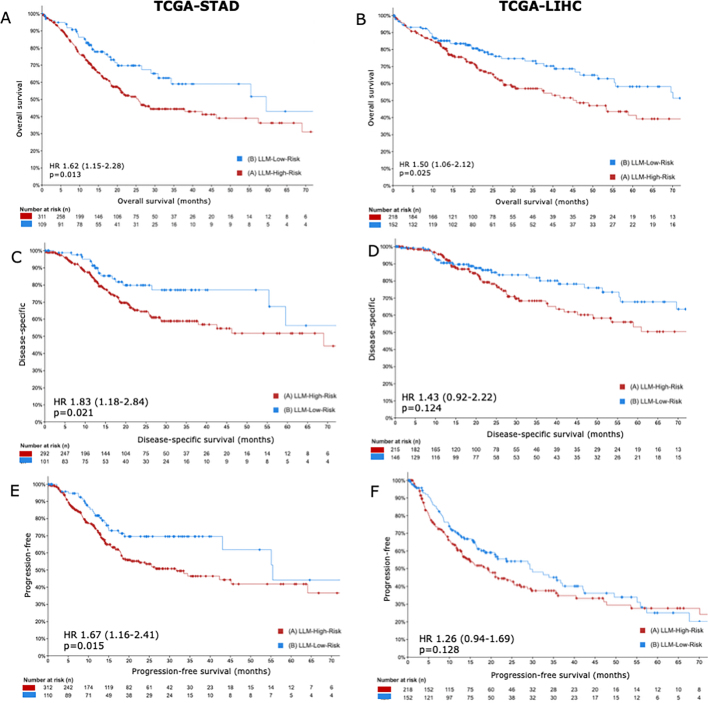
LLM-Risk Score can predict survival in other gastrointestinal cancers. Kaplan-Meier curves analysis for the TCGA cohorts stomach adenocarcinoma (STAD) and liver hepatocellular carcinoma (LIHC) for (A-B) Overall Survival (OS), (C-D) Disease specific Survival (DSS) and (E-F) Progression Free Survival (PFS) stratified by LLM High-risk and LLM Low-risk patients.

We quantified how often key prognostic elements were explicitly documented in the pathology report. In CRC, pT, pN, and pM were present in nearly all reports, with 99.8%, 99.8%, and 100.0% of cases, respectively. In contrast, detailed morphological and molecular markers were largely absent: tumor budding appeared in only 0.2% of reports, LVI in 9.5%, PNI in 1.7%, and MSI/MMR status in only 4.1%. Margin status was documented in 53.8% of CRC reports (Table 1). Similar patterns were observed in STAD and LIHC, where TNM staging elements were consistently present, but morphological and molecular markers (e.g., tumor budding, MSI) remained largely undocumented. We evaluated the ability of the LLM-derived risk scores to stratify survival in patients with CRC from the TCGA dataset (*n* = 613). The LLM stratified *n* = 313 patients into high-risk and *n* = 300 patients into low-risk groups based solely on their unstructured pathology reports (Fig. 1A). Kaplan–Meier analysis demonstrated significantly worse survival outcomes in the LLM-based high-risk group across all endpoints: OS, PFS, and DSS. Specifically, high-risk patients had significantly shorter OS (HR = 2.77, 95% CI: 1.92–3.97, *p* < 0.001; Fig. 1B), PFS (HR = 2.93, 95% CI: 2.11–4.08, *p* < 0.001; Fig. 1C), and DSS (HR = 5.85, 95% CI: 3.66–9.36, *p* < 0.001; Fig. 1D). Multivariate Cox proportional hazards analysis confirmed that the LLM-generated risk category remained independently associated with worse survival for PFS after adjusting for clinical covariates such as age, gender, MSI status, pT, pN, and positive nodal counts, whereas no independent association was observed for OS and DSS (Fig. 1E–G). To assess the added prognostic value of the LLM-derived risk score, we compared a clinical-only model against a combined model further incorporating the LLM risk category. The clinical-only model demonstrated good discrimination for PFS (C-Index = 0.688), OS (C-Index = 0.696), and DSS (C-Index = 0.747). Adding the LLM-derived risk group consistently improved model performance across all endpoints (PFS: C-Index = 0.701, Δ = +0.013; OS: C-Index = 0.702, Δ = +0.006; DSS: C-Index = 0.752, Δ = +0.005), reaching statistical significance for PFS in a likelihood ratio test (*p* = 0.026). Consistent with these findings, OS was significantly worse in the LLM-derived high-risk group in STAD and LIHC cohorts with a HR of 1.62 (95% CI: 1.15–2.28, *p* = 0.013; Supplementary Fig. 2A) and HR of 1.50 (95% CI: 1.06–2.12, *p* = 0.025; Supplementary Fig. 2B), respectively. DSS and PFS were additionally significant in STAD (HR = 1.83, 95% CI: 1.18–2.84, *p* = 0.021, and HR = 1.67, 95% CI: 1.16–2.41, *p* = 0.015) but not in LIHC (Supplementary Fig. 2). In all models, LLM risk stratification had comparable prognostic value compared to traditional clinical variables.

### LLM reasoning reflects clinically relevant prognostic features

**Supplementary figure 3:**
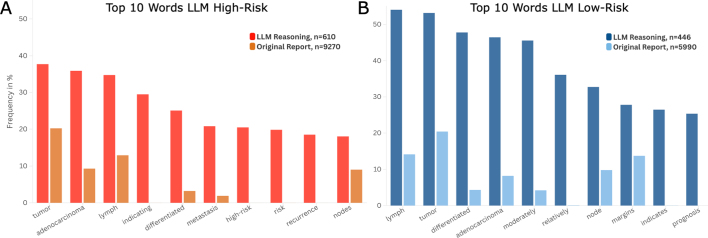
Word Frequency Analysis of LLM Reasoning OutputsTop 10 words from the LLM Reasoning in relative frequency in % compared to occurrence of the word relative frequency in % in the original Pathological Report for (A) LLM High-risk and (B) LLM Low-Risk.

To qualitatively explore how the LLM arrives at the assigned risk categories, we looked at the free-text rationales for representative CRC cases (Fig. 2A–B). In high-risk predictions, the rationales referenced adverse features such as the presence of “multiple positive lymph nodes,” or “vascular invasion,” and poor histological differentiation, often integrating several co-occurring findings into a single risk assessment. In contrast, low-risk predictions commonly emphasized “well-differentiated” tumors, “small tumor” confined to the bowel wall, the absence of nodal involvement, or “negative surgical margins”. To complement this qualitative assessment, we analyzed the most frequent words in the model's free-text reasoning field for CRC and compared them with word frequencies in the original report (Supplementary Fig. 3). The most frequent words in the LLM high-risk group were tumor, adenocarcinoma, and lymph, whereas in the LLM low-risk group, lymph, tumor, and differentiated were most common. The distribution of these terms between the original pathology reports and the LLM's reasoning output also highlighted that the model emphasizes specific high-yield features, for example, metastasis appearing in both the original report and LLM rationale in high-risk cases. It is important to note that this analysis focused solely on the presence of individual words, without capturing negations or the broader contextual meaning. For example, terms such as invasion could appear in low-risk cases in the context of phrases like no invasion, highlighting that word frequency alone does not fully reflect the model's nuanced interpretation of the text. High-risk predictions were associated with terms such as advanced, aggressive, vascular, infiltration, and invasive, whereas low-risk predictions frequently included distant, favorable, lower, small, and better (Fig. 2C). The distribution of pathology report lengths was broadly similar between the two LLM risk groups, with substantial overlap across the full range of word counts. Although reports classified as LLM high-risk tended to be slightly longer on average, the difference was not sufficient to clearly separate the cohorts (see Fig. 2D). The median word count for the high-risk group was 220 words (range: 30–3352), compared with a median of 134 words for the low-risk group (range: 26–1904). Overall, whereas LLM high-risk reports showed a mild tendency toward longer texts, report length alone does not appear to be a strong or singular discriminator for the LLM's risk classification.

### Correlation between LLM risk score and clinicopathological variables

To further explore the relationship between LLM predictions and conventional clinical variables, we computed a correlation matrix (Fig. 2E). LLM risk scores were moderately correlated with AJCC stage (*ρ* = 0.59), pN stage (*ρ* = 0.56), and pT stage (*ρ* = 0.32), but showed minimal correlation with patient age (*ρ* = −0.11) and gender (*ρ* = −0.05). These findings indicate that, whereas the model captures information related to established staging criteria, likely already reflected in the report, it also incorporates additional characteristics that are not explicitly mentioned and may not be included in the report at all. By integrating these extra signals, the model can provide a more nuanced assessment, potentially enhancing prognostic accuracy.

## Discussion

Pathology reports are exceptionally information-dense. Expert pathologists are trained to recognize prognostically meaningful tumor features and systematically describe a cancer's phenotype in their report and architecture, including invasive growth patterns, inflammatory infiltrates, stroma reaction patterns and routes of spread (lymphovascular/perineural invasion), often with margin and adjacent-tissue context.^15^, ^16^ Many different histopathological biomarkers (such as grade of differentiation, morphological subtyping, staging, tumor budding in CRC, perineural/lymphovascular invasion, and desmoplasia patterns) have been identified and refined by expert pathologist over the last decades and solely rely on H&E histopathology.^17^ Hence, these elements are embedded in histopathology reports and therefore, available for almost every cancer patient and increasingly also in a standardized structured form (e.g., TNM classification) used for clinical decision-making. Despite these advances, in everyday practice, reports still vary by institution, subspecialty, and reporting template, and not all potentially relevant details are consistently encoded as discrete variables. Narrative microscopic and macroscopic descriptions, therefore, continue to contain prognostic information that is not directly accessible to conventional risk models. To bridge this gap, we applied an open-source locally available LLM to distill unstructured pathology text of three different TCGA cohorts into a single, reproducible survival risk score, unlocking explicitly mentioned but also potential latent signals that pathologists already document. By doing so, our results show that a binary LLM-derived risk score has significant prognostic power. In CRC, this score modestly improved the discriminative performance of a model based on clinical covariates alone. This suggests that LLMs are capable of interpreting pathologist language at a semantic- and contextual level, capturing nuances such as the severity of invasion patterns, accompanying stromal or inflammatory reactions, multi-sentence context cues that are embedded in the prose and are currently not always fully reflected in the set of encoded variables and may therefore, only partially enter downstream prognostic and predictive assessment—even though pathologists invest substantial time, expertise, and attention to assess and describe these morphological features.

Clinically, our findings imply that LLM-generated risk scores could complement established staging systems by providing immediate, automated, and reproducible risk estimates. In contrast to other proposed risk stratifications in the investigated entities, such as molecular subtyping, LLM-based risk stratification only incurs minimal additional costs.

These results also align with broader trends toward multimodal oncology models that integrate text, imaging, and genomics.^18^, ^19^ Because pathology reports summarize microscopic findings that are themselves strongly prognostic, LLM-derived scores may serve as an efficient extra representation for downstream multimodal models, particularly in institutions, where digital pathology imaging is not yet widely available.

### Limitations

Even though we provide evidence here that unstructured pathology reports contain relevant and potentially additional prognostic information, our study has of course several limitations. First of all, pathology reports are quite diverse, and the performance of the model may drop with differences in report styles, due to different languages (all TCGA reports are in English), reporting templates or “department styles.” Nevertheless, the TCGA datasets were collected across different centers, indicating at least some degree of generalizability. Additionally, many TCGA reports predate widespread synoptic standardization, which likely explains the low documentation rates of tumor budding and MSI/MMR status. In contrast, pathology slide-based image models^20^, ^21^ do not depend on those stylistic shifts. Mitigations for these local differences include multisite external validation, prompt/model domain adaptation (e.g., few-shot tuning on local reports), and reporting calibration and performance by site/language. Second, narrative bias is possible. Knowing that a tumor is deeply invasive (e.g., pT3/4) can “color” the pathologist's description and lead to overemphasizing aggressive patterns (e.g., some pathologists may describe high budding and extensive LVI if they know a tumor has already relapsed or shown vascular or nodal metastases), creating confirmation bias that the LLM can inadvertently learn. Thirdly, we only investigated three common tumor types arising in the digestive system (CRC, STAD, and HCC) in our current study. Moreover, several of our explainability analyses should be viewed as exploratory and descriptive. These approaches are a qualitative attempt to gain insights into how the LLM derived risk scores. Finally, the LLM-derived risk score is restricted to information contained in pathology reports and cannot capture unreported or visually imperceptible biomarkers, which may be detectable by models trained directly on whole-slide images. Future studies are necessary to test if our findings are also valid for tumors outside of the digestive tract and rarer tumor types, sometimes even showing a larger morphological heterogeneity such as sarcomas. Future work should explore fine-tuning and externally validating local LLMs on larger state-of-the-art pathology corpora, integrating chain-of-thought rationales for interpretability, and comparing text-derived risk signals with those obtained from digital pathology image analysis.

### Outlook

This study is an initial proof-of-concept that LLMs can unlock prognostic signals from unstructured pathology reports. Next steps should include prospective, multisite external validation across languages and report templates, with prespecified endpoints and robustness checks (e.g., prompt ablation) to demonstrate clinical utility. Biologically, linking the LLM risk score to orthogonal omics (DNA, RNA, and/or protein level) and treatment response will test whether the text-derived signal maps to actionable tumor biology.

## Conclusion

LLMs can extract prognostic information from narrative pathology reports and convert it into binary survival biomarkers with independent predictive value beyond conventional staging systems. This approach recovers expert knowledge currently lost in unread prose while requiring no additional tissue analysis, molecular testing, or pathologist workload. Using open-source, locally deployable models ensures fast clinical feasibility and privacy preservation, offering a practical strategy to enhance risk stratification for treatment decision-making.

## Ethic statement

This study was carried out in accordance with the Declaration of Helsinki. The collection of the tissue samples for the training cohort FOXTROT-CRC was granted by the Northern and Yorkshire Research Ethics Committee (Jarrow, UK; Unique Reference Number: 07/MRE03/24). The analysis of the first testing cohort, CR07-CRC, was approved in the UK by the Multicentre Research Ethics Committee for Scotland (ISRCTN study registration number 28785842). The analysis of the second testing cohort DACHS-CRC (an epidemiological study which is led by the German Cancer Research Center, DKFZ, Heidelberg, Germany) was approved by the ethics committee of the Medical Faculty, University of Heidelberg under 310/2001.

## CRediT authorship contribution statement

Chiara M. L. Loeffler: Conceptualization, Data curation, Investigation, Visualization, Writing – original draft, Writing – review & editing. Nic G. Reitsam: Formal analysis, Investigation, Writing – review & editing. Fabian Wolf: Data curation, Software, Writing – review & editing. Esther H. Stueker: Visualization, Formal analysis, Writing – review & editing. Hannah S. Muti: Investigation, Writing – review & editing. Jakob Nikolas Kather: Supervision, Resources, Validation, Writing – review & editing. Isabella C. Wiest: Conceptualization, Data curation, Supervision, Validation, Writing – review & editing.

## Funding

**JNK** is supported by the 10.13039/501100005972German Cancer Aid DKH (DECADE, 70115166), the German Federal Ministry of Research, Technology and Space BMFTR (PEARL, 01KD2104C; CAMINO, 01EO2101; TRANSFORM LIVER, 031L0312A; TANGERINE, 01KT2302 through ERA-NET Transcan; Come2Data, 16DKZ2044A; DEEP-HCC, 031L0315A; DECIPHER-M, 01KD2420A; NextBIG, 01ZU2402A), the 10.13039/100005930German Research Foundation DFG (TRR 412/1, 535081457; SFB 1709/1 2025, 533056198), the 10.13039/501100001655German Academic Exchange Service DAAD (SECAI, 57616814), the German Federal Joint Committee G-BA (TransplantKI, 01VSF21048), the 10.13039/501100000780European Union EU's Horizon Europe research and innovation programme (ODELIA, 101057091; GENIAL, 101096312), the 10.13039/501100000781European Research Council ERC (NADIR, 101114631), the 10.13039/100001006Breast cancer Research Foundation (BELLADONNA, BCRF-25-225) and the National Institute for Health and Care Research NIHR (10.13039/100014461Leeds Biomedical Research Centre, NIHR203331). The views expressed are those of the author(s) and not necessarily those of the NHS, the NIHR, or the Department of Health and Social Care. This work was funded by the 10.13039/501100000780European Union. Views and opinions expressed are, however, those of the author(s) only and do not necessarily reflect those of the European Union. Neither the European Union nor the granting authority can be held responsible for them. **NGR** is supported by the clinician scientist programme of the Faculty of Medicine, University of Augsburg, Germany.

## AI statement

Example: In accordance with the COPE (Committee on Publication Ethics) position statement of 13 February 2023 (https://publicationethics.org/cope-position-statements/ai-author), the authors hereby disclose the use of the following artificial intelligence models during the writing of this article. GPT-4o (OpenAI) for checking spelling and grammar.

## Declaration of competing interest

The authors declare the following financial interests/personal relationships which may be considered as potential competing interests:

Jakob Nikolas Kather reports financial support was provided by German Cancer Aid DKH. Jakob Nikolas Kather reports financial support was provided by German Federal Ministry of Research, Technology and Space BMFTR. Jakob nikolas Kather reports financial support was provided by German Research Foundation DFG. Jakob Nikolas Kather reports financial support was provided by German Academic Exchange Service DAAD. Jakob Nikolas Kather reports financial support was provided by German Federal Joint Committee G-BA. Jakob Nikolas Kather reports financial support was provided by European Union Horizon Europe research and innovation programem. Jakob nikolas Kather reports financial support was provided by European Research Council ERC. Jakob Nikolas Kather reports financial support was provided by Breast Cancer Research Foundation. Jakob Nikolas Kather reports financial support was provided by National Institute for Health and Care Research NIHR. Chiara M.L. Loeffler reports a relationship with AstraZeneca that includes: speaking and lecture fees. Chiara M. L. Loeffler reports a relationship with AbbVie Inc. that includes: speaking and lecture fees. Nic G. Reitsam reports a relationship with Bruker Spatial Biology Inc. that includes: travel reimbursement. Isabella Wiest reports a relationship with AstraZeneca that includes: speaking and lecture fees. Jakob Nikolas Kather reports a relationship with AstraZeneca that includes: consulting or advisory. Jakob Nikolas Kather reports a relationship with Biooptimus that includes: consulting or advisory. Jakob Nikolas Kather reports a relationship with StratifAI that includes: equity or stocks. Jakob Nikolas Kather reports a relationship with Synagen that includes: equity or stocks. Jakob Nikolas Kather reports a relationship with Spira Labs that includes: equity or stocks. Jakob Nikolas Kather reports a relationship with AstraZeneca that includes: speaking and lecture fees. Jakob Nikolas Kather reports a relationship with Bayer Corporation that includes: speaking and lecture fees. Jakob Nikolas Kather reports a relationship with Daiichi Sankyo Inc. that includes: speaking and lecture fees. Jakob Nikolas Kather reports a relationship with Eisai Inc. that includes: speaking and lecture fees. Jakob Nikolas Kather reports a relationship with Janssen Pharmaceuticals Inc. that includes: speaking and lecture fees. Jakob Nikolas Kather reports a relationship with Merck & Co Inc. that includes: speaking and lecture fees. Jakob Nikolas Kather reports a relationship with MSD that includes: speaking and lecture fees. Jakob Nikolas Kather reports a relationship with Bristol Myers Squibb Co that includes: speaking and lecture fees. Jakob Nikolas Kather reports a relationship with Roche that includes: speaking and lecture fees. Jakob Nikolas Kather reports a relationship with Pfizer that includes: speaking and lecture fees. Jakob Nikolas Kather reports a relationship with Fresenius that includes: speaking and lecture fees. If there are other authors, they declare that they have no known competing financial interests or personal relationships that could have appeared to influence the work reported in this article.

**CMLL** reports honoraria from AstraZeneca and Abbvie. **NGR.** received compensation for travel expenses from Bruker Spatial Biology. **ICW**. received honoraria from AstraZeneca. All the other authors report nothing to declare. **JNK.** declares ongoing consulting services for AstraZeneca and Bioptimus. Furthermore, he holds shares in StratifAI, Synagen, and Spira Labs, has received an institutional research grant from GSK and AstraZeneca, as well as honoraria from AstraZeneca, Bayer, Daiichi Sankyo, Eisai, Janssen, Merck, MSD, BMS, Roche, Pfizer, and Fresenius.

## Appendix A Supplementary data

Supplementary Table 1: LLM-Generated Risk Classifications by Patient ID. Individual risk assessment results for the all three TCGA cohorts used in this study. This table. Provides the full list of patients and their LLM-assigned risk scores, representing the initial cohort before clinical data filtering.

Supplementary Table 2: Comparative Word Frequency Analysis of LLM Reasonings and Original Pathology Reports. This table details the most frequently occurring words within the LLM Reasoning Field and the Original Pathology Reports, stratified by risk level. The left columns present frequency data for high-risk (total word count: 610) and low-risk (total word count: 446) LLM classifications. The right columns display the corresponding word frequencies from the source pathology reports for high-risk (total word count: 9270) and low-risk (total word count: 5990) cases. Each entry lists the total number of times a specific term appeared.
